# Proteomic Analysis of Niemann-Pick Type C Hepatocytes Reveals Potential Therapeutic Targets for Liver Damage

**DOI:** 10.3390/cells10082159

**Published:** 2021-08-21

**Authors:** Elisa Balboa, Tamara Marín, Juan Esteban Oyarzún, Pablo S. Contreras, Robert Hardt, Thea van den Bosch, Alejandra R. Alvarez, Boris Rebolledo-Jaramillo, Andres D. Klein, Dominic Winter, Silvana Zanlungo

**Affiliations:** 1 Departamento de Gastroenterología, Facultad de Medicina, Pontificia Universidad Católica de Chile, Santiago 8330024, Chile; tlmarin@uc.cl (T.M.); jeoyarzu@uc.cl (J.E.O.); 2 Cell and Developmental Biology Center, National Heart, Lung, and Blood Institute, National Institutes of Health, Bethesda, MD 20892-8018, USA; pablo.contrerassoto@nih.gov; 3 Institute for Biochemistry and Molecular Biology, Medical Faculty, University of Bonn, 53115 Bonn, Germany; rhardt@uni-bonn.de (R.H.); t.f.h.van.den.bosch@gmail.com (T.v.d.B.); 4 Laboratory of Cell Signaling, Department of Cellular and Molecular Biology, Biological Sciences Faculty, CARE UC, Pontificia Universidad Católica de Chile, Santiago 8330024, Chile; aalvarez@bio.puc.cl; 5 Centro de Genética y Genómica, Facultad de Medicina, Clínica Alemana Universidad del Desarrollo, Santiago 7710162, Chile; brebolledo@udd.cl (B.R.-J.); andresklein@udd.cl (A.D.K.)

**Keywords:** liver damage, Niemann Pick type C disease, lysosomal storage disorder, proteomic analysis, mass spectrometry

## Abstract

Niemann-Pick type C disease (NPCD) is a lysosomal storage disorder caused by mutations in the *NPC1* gene. The most affected tissues are the central nervous system and liver, and while significant efforts have been made to understand its neurological component, the pathophysiology of the liver damage remains unclear. In this study, hepatocytes derived from wild type and *Npc1*^−/−^ mice were analyzed by mass spectrometry (MS)-based proteomics in conjunction with bioinformatic analysis. We identified 3832 proteins: 416 proteins had a *p*-value smaller than 0.05, of which 37% (*n* = 155) were considered differentially expressed proteins (DEPs), 149 of them were considered upregulated, and 6 were considered downregulated. We focused the analysis on pathways related to NPC pathogenic mechanisms, finding that the most significant changes in expression levels occur in proteins that function in the pathways of liver damage, lipid metabolism, and inflammation. Moreover, in the group of DEPs, 30% (*n* = 47) were identified as lysosomal proteins and 7% (*n* = 10) were identified as mitochondrial proteins. Importantly, we found that lysosomal DEPs, including CTSB/D/Z, LIPA, DPP7 and GLMP, and mitocondrial DEPs, AKR1B10, and VAT1 had been connected with liver fibrosis, damage, and steatosis in previous studies, validiting our dataset. Our study found potential therapeutic targets for the treatment of liver damage in NPCD.

## 1. Introduction

Niemann Pick type C disease (NPCD) is a neurovisceral lysosomal storage disorder (LSD) with an estimated incidence of 1/90,000 live births [1]. It is caused by mutations in the *NPC1* or *NPC2* genes, which encode lysosomal cholesterol transport proteins that mediate free cholesterol efflux from this compartment. Therefore, NPC1 or NPC2 deficient cells accumulate cholesterol in lysosomes. The most affected tissues are the central nervous system (CNS) and the liver [2]. The symptoms and clinical presentation are extremely heterogeneous, with age of onset ranging from the perinatal period to adulthood. Visceral involvement includes the liver, spleen, and sometimes the lungs, while possible neurological disorders consist mainly of cerebellar ataxia, dysarthria, dysphagia, and progressive dementia. The onset of systemic symptoms frequently precedes the start of neurological symptoms [3].

Although most patients with NPCD die from complications of the neurological disorder [3], some also develop fatal liver disease [4]. Aside from liver failure [5,6], NPC patients frequently present with neonatal jaundice (52%), enlargement of the spleen (36%), liver (31%), and ascites (19%) [7]. At the organ and tissue levels, mice lacking the NPC1 protein (NPC mice) develop liver disease with hepatomegaly, cell death, infiltration of foamy macrophages, inflammation, proliferation of hepatic stellate cells, and fibrosis [8,9,10].

Usually, the systemic disease is not very severe, except for the perinatal period in which a small subset of patients die in the first six months of life [3]. Given the hepatic manifestations in the development of NPCD and to design treatments for liver symptoms that will provide a better quality of life for patients, it is necessary to understand the molecular pathways that are affected in NPC liver cells.

Proteomics technology is a tool that allows full-scale analysis of all proteins involved in a pathological process. It can provide disease-related protein detection by comparing protein expression profiles between normal and pathological samples. The detection of these disease-related proteins or biomarkers can be used to monitor the activity and development of the disease, and its prognosis [11]. Moreover, the data obtained from the proteomic analysis can be used for dissecting the molecular targets and signaling pathways of a specific pathology. Previously, mass spectrometry (MS)-based proteomics protocols were applied to the investigation of different LSDs models such as CLN1 disease [12], Gaucher disease [13] and Fabry disease [14], leading to significant advances in the understanding of lysosomal function and pathology [15]. Specifically, OMICs analyses have been successfully used in a variety of studies to identify disease-related changes in NPCD. Investigation of the mannose 6 phosphate-modified brain proteome of NPC1 and NPC2 knock-out (KO) mice revealed similar patterns of altered lysosomal hydrolases expression levels for both genes [16]. Furthermore, proteomic and lipidomic analyses of lysosomes and plasma membranes from NPC1 KO HeLa cells yielded insights into alterations in the composition of specific subcellular compartments [17]. Another study that investigated changes in the lipidome of liver samples from NPC1 KO mice showed changes in the accumulation of various types of membranes and storage lipid species [18]. Alterations of the lysosomal N-glycome due to NPCD were also addressed. Using MS-based approaches, the authors showed that high mannose and sialylated N-glycans were altered, with a potential role in the lysosomal impairments observed in NPCD [19]. Interestingly, previous reports have already shown differential liver proteome in the NPC1 mouse model using whole liver tissue [20,21]. The hepatocytes are the main cells involved in liver metabolism, and account for approximately 78% of liver tissue volume [22,23]. Moreover, it has been described that hepatocyte apoptosis may be a primary cause of liver dysfunction and liver failure in NPC murine models [9,10]. In addition, restoring NPC1 into hepatocytes, but not Kupffer cells, is sufficient to reverse NPC liver damage in mice [24]. These results suggests that the hepatocyte is the cell type that should be therapeutically targeted. Therefore, we are interested in studying the proteome of hepatocytes in NPCD liver pathology.

In order to investigate the pathophysiology underlying liver damage, specifically the contribution of the hepatocyte in NPCD, we performed MS-based proteomic analyses to compare the protein composition of primary hepatocyte cultures from wild type (WT) and NPC1 deficient (*Npc1^−/−^)* mice. We found 155 differentially expressed proteins (DEPs): 30% (*n* = 47) were identified as lysosomal proteins and 7% (*n* = 10) as mitochondrial proteins. Interestingly, lysosomal proteins such as cathepsin B (CTSB), lysosomal acid lipase (LIPA), dipeptidyl peptidase 2 (DPP), and glycosylated lysosomal membrane protein (GLMP), which are known to be involved in liver fibrosis, liver damage, and steatosis, were found to be differentially expressed.

Our quantitative proteomic approach using *Npc1^−/−^* primary culture hepatocytes reveals consistent changes in lysosomal and mitochondrial proteins, providing information on potentially interesting therapeutic targets for the development of treatments for damaged liver in NPCD.

## 2. Materials and Methods

### 2.1. Primary Mouse Hepatocytes Isolation

Primary mouse hepatocytes were isolated as described previously [25]. Briefly, hepatocytes from male BALB/c *Npc1^+/+^* (WT) and *Npc1^−/−^* mice (eight weeks old) were isolated by collagenase perfusion at a flow rate of 7–9 mL/min and cultured in DMEM/F12 media (Gibco, Paisley, UK; # 21331-020) supplemented with 10% FBS (Biowest; Riverside, MO, USA; # S1810-500) for the first 3 h post isolation, together with 10,000 U/mL Penicillin-Streptomycin (Gibco, Gran Island, NY, USA; # 15140-122), 200 mM L-Glutamine (Gibco, Paisley, UK; # 25030-024), 7.5 mM D-Glucose (Sigma, St. Louis, MO, USA) and 150 mM Hepes pH 7.4 (Sigma, St. Louis, MO, USA). For subsequent treatments, FBS was replaced with 1 mM methionine (Sigma, St. Louis, MO, USA). After 3 h, the medium was changed to warm maintenance medium. The hepatocytes were kept in a humidified CO_2_ incubator for 16–20 h. Then, hepatocytes were washed with PBS and trypsinized and the pellet stored at −80 °C until further use.

### 2.2. Proteomic Sample Preparation

Samples were processed by in-solution digestion using RapiGest (Waters, Milford, MA, USA) as described before [26], with minor adjustments. Briefly, WT and *Npc1^−/−^* cells (three replicates each) were lysed in lysis buffer (50 mm HEPES, 150 mm KCl, 1 mm MgCl_2_, 10% Glycerol, 1% Triton-X 100) supplemented with protease inhibitors using a sonifier (ultrasonic homogenizer UP50H, Hielscher, Teltow, Germany). Lysates were then cleared by centrifugation and protein concentration was measured using the DC protein assay (Biorad, Hercules, CA, USA). For each sample, 130 µg of protein were acetone precipitated and the pellets dissolved in 0.1% RapiGest, 100 mM TEAB (Sigma, St. Louis, MO, USA). Disulfide bonds were reduced with 5 mM DTT (Sigma, St. Louis, MO, USA) at 56 °C for 45 min, alkylated with 20 mM acrylamide (Sigma, St. Louis, MO, USA) at RT for 30 min. The reaction was quenched by the addition of 5 mM DTT [27]. Finally, trypsin (Promega, Madison, WI, USA) was added at an enzyme to sample ratio of 1:100, the volume adjusted to 130 µL, and samples digested in solution at 37 °C overnight. The following day, the resulting peptides were labeled with TMT6plex reagents (Thermo Fisher Scientific, Waltham, MA, USA): labels were dissolved in 41 µL acetonitrile, added to the samples, and incubated at room temperature for 1.5 h. After quenching with 8 µL of 5% hydroxylamine (Sigma, St. Louis, MO, USA) at RT for 15 min, samples were combined and the ACN content was reduced by vacuum centrifugation to <1%. Subsequently, RapiGest was removed via acidic cleavage (5% TFA final, 37 °C, 45 min) and precipitation by centrifugation (20,000 g, RT, 30 min). The combined peptide digest was subjected to desalting using 1cc Oasis HLB solid-phase extraction cartridges (Waters, Milford, MA) and the eluate fraction dried by vacuum centrifugation. For strong anion exchange (SAX) fractionation, the sample was resuspended in SAX resuspension buffer (20 mM acetic acid, 20 mM phosphoric acid, 20 mM boric acid, pH 11) and 100 µg of peptides were fractionated into six fractions (pH 11, 8, 6, 5, 4, 3) using SAX tip columns as described before [28]. Fractions were desalted using C18 StageTips [29] and eluates dried by vacuum centrifugation.

### 2.3. LC-MS/MS Analysis

Dried peptide samples were dissolved in 10 µL 5% formic acid, 5% ACN and subjected to LC-MS analysis using a Dionex UltiMate 3000 RSLCnano coupled with an Orbitrap Fusion Lumos (both Thermo Fisher Scientific, Bremen, Germany). We used 40 cm long analytical columns produced in-house as follows: spray tips were generated from 100 µm inner diameter/360 µm outer diameter fused silica capillaries with a P-2000 laser puller (Sutter Instrument, Novato, CA, USA) and packed with reversed-phase material (1.9 µm ReproSil-Pur 120 C18-AQ, Dr. Maisch, Ammerbuch-Entringen, Germany). Samples (2.5 µL) were loaded for 25 min onto the analytical column at a flow rate of 600 nL/min using 99% solvent A (0.1% FA in H_2_O) and 1% solvent B (90% ACN, 0.1% FA). Afterward, peptides were eluted using a linear gradient of 1–40% solvent B for 120 min at a flow rate of 300 nL/min. The mass spectrometer was operated in the positive ion mode with a capillary voltage of 2.1 kV. Survey spectra were acquired in the Orbitrap mass analyzer from m/z 375–1500 at a resolution of 60,000 with automated gain control (AGC) of 4 × 10^5^, maximal injection time of 50 ms, and resonance frequency of 30%. Precursors with a charge ≥ 2 and an intensity ≥ 5 × 10^4^ were isolated in the quadrupole (width: 1.4 m/z) and subjected to higher collisional dissociation fragmentation (HCD, normalized collision energy of 30) using the top speed mode (cycle time: 3 s). Fragment spectra were acquired in the Orbitrap at a resolution of 15,000 and the mass range was determined automatically with a defined low mass of m/z 110. The AGC was set to 5 × 10^4^ and the maximum injection time to 60 ms. Dynamic exclusion was enabled (exclusion time: 60 s, mass tolerance: +/− 10 ppm).

### 2.4. Data Analysis

Thermo raw files were processed using the Proteome Discoverer software package (version 2.4, Thermo Fisher Scientific, Waltham, MA, USA). First, mass spectra were recalibrated using the SpectrumRC node, followed by peptide identification via a two-step Mascot (version 2.6.1, Matrix Sciences) search against Swiss-Prot (560,459 entries, downloaded 2019-06) restricted to the taxonomy *Mus musculus* in combination with two databases containing common contaminants: cRAP (Available online: https://reprint-apms.org/, accessed on 2 January 2015) and MaxQuant contaminants (Available online: https://www.maxquant.org/, accessed on 13 December 2019). In the first pass search, enzyme specificity was set to trypsin/p, with up to two missed cleavages, and 10 ppm/20 mmu precursor/fragment tolerance. The following modifications were enabled: variable: oxidation (M), acetyl (protein N-term); fixed: propionamide (C), TMT6plex (K and peptide N-term). Subsequently, peptide spectral matches (PSM) were validated by Percolator, and spectra with a false discovery rate (FDR) value > 0.01 were subjected to a second pass search using relaxed search settings (enzyme: semiTrypsin, missed cleavages: 1, variable modifications: oxidation (M), acetyl (protein N-term), propionamide (C), TMT6plex (K and peptide N-term)). PSMs from the second pass search were also validated by Percolator. Identifications from both searches were aggregated to peptide and protein groups following the strict parsimony principle and filtered at 0.01 FDR. TMT reporter ions were quantified with the corresponding node using the following settings: peak integration tolerance: 20 ppm, peak integration method: most confident centroid, MS order: 2, reporter ion abundance based on: automatic, co-isolation threshold: 50%, average reporter S/N: 10. For protein quantification, unique and razor peptides were used and protein abundances of each labeling channel were further normalized based on the total peptide amount. After removal of proteins marked as contaminants, the resulting master protein table was exported and statistical analysis of differentially regulated proteins between WT and KO was performed using Perseus (version: 1.6.14, Available online: https://maxquant.net/perseus/, accessed on 19 July 2020). For this, proteins were first filtered for entries having at least two expression values in one genotype, followed by a two-sample t-test. Proteins exhibiting a *p*-value < 0.05 and a log_2_ fold change > 1 or < −1 were considered to be significantly changed. The mass spectrometry proteomics data have been deposited to the ProteomeXchange Consortium via the PRIDE [30] partner repository with the dataset identifier PXD026623 and 10.6019/PXD026623.

### 2.5. Upstream Regulator Analysis

Data were analyzed through the use of IPA (QIAGEN Inc., Available online: https://www.qiagenbioinformatics.com/products/ingenuity-pathway-analysis, accessed from 13 June 2020 through 12 January 2021) [31]. The *p*-value of overlap measures whether there is a statistically significant overlap between the dataset molecules and those regulated by an upstream regulator and is calculated using Fisher’s Exact Test, and significance is attributed to *p*-values < 0.05. The categories of the proteins were generated through the use of PANTHER. (Available online: pantherdb.org, accessed from 13 July 2020) [32].

### 2.6. Western Blot Analysis

Livers from six-week-old WT and *Npc1^−/−^* mice were homogenized in RIPA buffer with the complete protease inhibitor cocktail (Thermo Scientific, Waltham, MA USA; # 78430). 12% SDS-PAGE was run and proteins were blotted into Midi nitrocellulose membranes ( Bio-rad, Hercules, CA, USA; #17001915). The primary antibodies used were: anti-NPC1 serum kindly donated by Dr. William Garver (University of Arizona, Tucson, AZ, USA), anti-CTSB (Cell Signaling Technology, Inc., Danvers, MA, USA; (D1C7Y) # 31718), anti- LAMP1 (Cell Signaling Technology, Inc., Danvers, MA, USA; (D2D11) # 9091), anti-StarD3 (Invitrogen, Waltham, MA, USA; PA1-562), and anti-GAPDH (Santa Cruz Biotechnology, Dallas, TX, USA; (6C5) # sc32233). Anti-rabbit IgG HRP-linked Antibody (Cell Signaling Technology, Inc., Danvers, MA, USA; # 7074) and Goat anti-mouse IgG (H+L) (Invitrogen, Waltham, MA, USA; #62-6520) secondary antibodies HRP conjugate were used to visualize proteins in the BOX Model Chemi XRQ Syngene (UK).

## 3. Results

### 3.1. NPC1 Deficient Hepatocytes Show 416 Proteins with Significant Expression Changes

**Figure 1:**
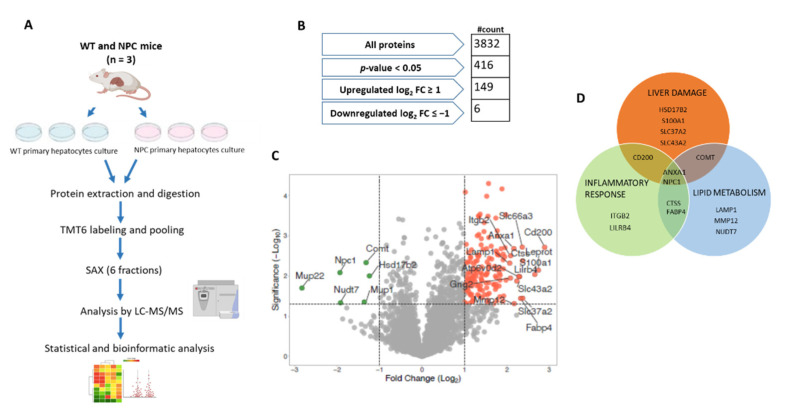
Proteomic analysis of a hepatocyte model of Niemann-Pick C disease. (**A**) Schematic representation of the experimental workflow. (**B**) Summary for the processing of data. (**C**) Volcano plot obtained from quantitative proteomics analysis. Red and green dots represent proteins that show a log_2_ fold change ≥ 1 and log_2_ fold change ≤ −1. Proteins tagged show log_2_ fold change ≥ 2 and log_2_ fold change ≤ −1. (**D**) Venn diagram depicting proteins tagged with a role in liver damage (orange circle), inflammatory response (green circle), and lipid metabolism (blue circle).

To dissect the proteome of liver cells in NPCD we cultured primary hepatocytes from WT and Npc1^−/−^ adult mice and analyzed them by MS-based proteomics in conjunction with bioinformatics analyses using Perseus, QIAGEN Ingenuity Pathway Analysis (IPA), and PANTHER (Figure 1A). First, we performed protein extraction and RapiGest-assisted digestion (three replicates per genotype), followed by sixplex tandem-mass tag (TMT6) labeling of individual samples. This strategy allowed the analysis of all samples in one single LC-MS run, thereby increasing the robustness and reproducibility of peptide identification and quantification. After pooling samples labeled with the individual channels, the combined sample was divided into six fractions via strong-anion exchange chromatography (SAX) to reduce sample complexity and increase peptide identification numbers. Individual fractions were analyzed by LC-MS/MS followed by a combined database search. With this approach, we reliably identified 111,514 PSMs, 43,811 peptide groups, and 4847 protein groups at 1% FDR. After stringent filtering (removal of contaminants and proteins with < 2 valid values in one genotype), 3832 proteins presented robust quantification. Figure 1A shows a schematic representation of the experimental workflow used. To identify proteins with differences in abundance between both genotypes, we performed a two-sample t-test. We obtained 416 proteins that presented a *p*-value smaller than 0.05. A list of the number of proteins, peptides, and spectra identified/quantified is given in Supplementary Table S1. Then, 37% (*n* = 155) were deemed differentially expressed proteins (DEPs) with 149 upregulated (log_2_ fold change ≥ 1) and 6 downregulated (log_2_ fold change ≤ −1) (Figure 1B, Supplementary Table S2).

Proteins identified and quantified with the criteria described above were used to generate a volcano plot where regulated proteins are displayed in different colors and tagged proteins (*n* = 21) have a log_2_ fold change > 2 or a log_2_ fold change < −1 (Figure 1C). These proteins were classified by locations and type as shown in Supplementary Table S3. Additionally, these 21 proteins that showed major changes, were analyzed using the IPA software to predict significantly associated diseases or functions. We found that the majority of these proteins (71%), such as fatty acid-binding protein 4 (FABP4), annexin A1 (ANXA1), integrin subunit beta 2 (ITGB2), nudix hydrolase 7 (NUD7), and catechol O-methyltransferase (COMT), were significantly associated with lipid metabolism, liver damage, and the inflammatory response (Figure 1D). The role of these proteins in NPC liver disease has not been studied. It would be interesting to investigate whether, together with the other proteins with altered expression patterns, they participate in the pathogenesis of the disease.

### 3.2. Differentially Expressed Proteins in NPC1 Deficient Hepatocytes Are Predominantly Related to Alterations in Metabolism and Liver Function

**Figure 2:**
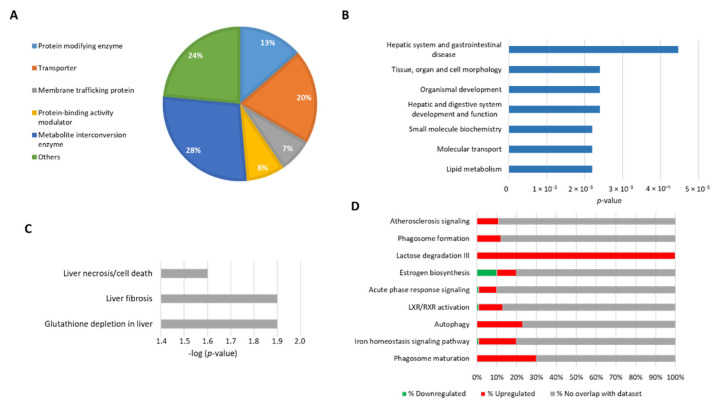
Disease and bifunctional analysis of the differential proteome of hepatocytes obtained from wild type and *Npc1 ^−/−^* mice. (**A**) Pie chart showing different functional classes of proteins based on the PANTHER database. (**B**) Top toxicity-related protein list from the sets of 155 altered proteins (upregulated and downregulated) that are known to be involved in a specific type of toxicity. Significance was determined using a right-tailed Fisher’s exact test to determine the probability of pathways from the IPA Knowledge Base Library of those most significantly enriched. (**C**) Top toxicity-related proteins list filtered by hepatotoxicity. (**D**) The bar chart represents the top canonical signaling pathways that were influenced by the NPC phenotype, *p*-values were determined using Fisher’s exact test with a threshold value of > 0.05. Ratios represent the percentage of genes from the data set that map to each canonical pathway showing those that are upregulated (in red) and downregulated (in green).

To place our experimental results within the context of biological functions we used the PANTHER Classification System (Available online: pantherdb.org, accessed from 13 July 2020). Considering 155 DEPs, our analysis revealed that 28% correspond to metabolic enzymes, 20% to transporters, and 13% to protein modifying enzymes (Figure 2A). Then, to elucidate significant correlations with pathological processes in which these proteins may participate, we used the IPA tool “Diseases and function”. We found that the most significant biological functions associated with our data were related to lipid metabolism, molecular transport, and hepatic system disease (Figure 2B). When we used the filter “Toxicity function” to identify changes related to hepatotoxicity, we found that many DEPs participate in liver fibrosis, necrosis, and depletion of glutathione (Figure 2C), confirming that NPC1 deficiency induces liver damage. This finding will help to attain a better understanding of the pathways that lead to liver damage in NPC disease.

Following the functional analysis, we determined the most affected canonical signaling pathways (Figure 2D). The stacked bars indicate the percentage of proteins from the data set that map to each canonical pathway, with green and red indicating downregulated and upregulated proteins, respectively. The open bars show proteins with no overlap in any canonical pathway. The pathway analysis indicates that many differentially expressed proteins belong to canonical pathways such as those involved in autophagy, phagosome maturation, and iron homeostasis. Our results are in agreement with previous reports showing that these processes (autophagy [21,33], phagosome maturation [34] and iron homeostasis) are altered in different NPC models [21,33,34,35].

### 3.3. Lysosomal and Mitochondrial Proteins Are Differentially Expressed in NPC1 Deficient Hepatocytes

**Figure 3:**
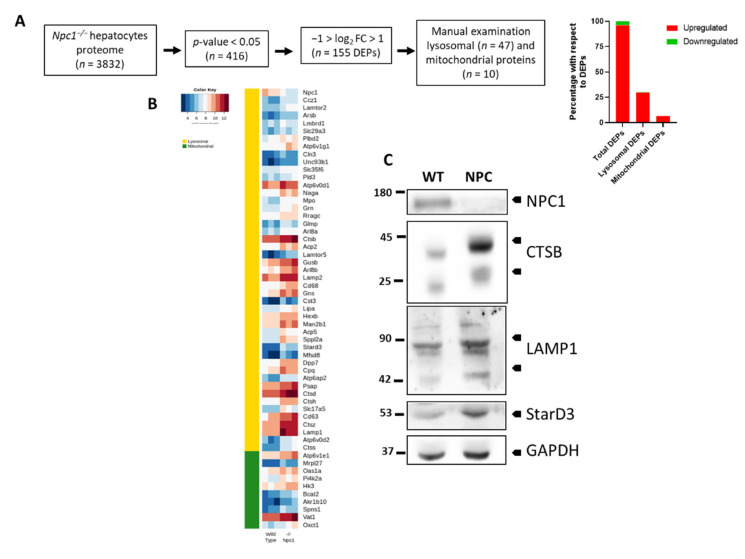
Lysosomal and mitochondrial proteins differentially expressed in hepatocytes from wild type and *Npc1^−/−^* mice. (**A**) Summary of the computational pipeline for proteome analysis and bar chart representation of the total, mitochondrial and lysosomal proteins that present changes in *Npc1^−/−^* hepatocytes. (**B**) Heat map presenting differentially expressed lysosomal and mitochondrial proteins in wild type and *Npc1^−/−^* hepatocytes (*n* = 3). (**C**) Western blot analysis of liver homogenates confirmed the decreased expression of NPC1 and increased expression of CTSB, LAMP1, and StarD3 proteins in 6 weeks old *Npc1^−/−^* mice (NPC) compared to wild type (WT) mice of the same age.

Since NPCD is a lysosomal lipid storage disorder and we and others have shown that there is also mitochondrial dysfunction associated with this pathology [36,37], we looked for lysosomal and mitochondrial proteins among the 155 DEPs. Figure 3A shows a summary of the proteome analysis computational pipeline used to find DEPs. We found that 30% of the DEPs were lysosomal proteins (*n* = 47) and 7% mitochondrial proteins (*n* = 10) (Figure 3A, Supplementary Table S4). Interestingly, all of these lysosomal (except NPC1) and mitochondrial proteins are overexpressed in *Npc1^−/−^* hepatocytes. Figure 3B shows the heat map of lysosomal and mitochondrial DEPs in WT and *Npc1^−/−^* hepatocytes. Next, we performed western blot experiments to investigate expression levels of proteins found to be altered in NPC hepatocytes by MS, which had been related to the pathogenesis of NPCD in the liver from *Npc1^−/−^* mice. This included cathepsin b (CTSB), lysosome-associated membrane glycoprotein 1 (LAMP1), and StAR-related lipid transfer protein 3 (STARD3) [38,39,40]. In agreement with the MS results, we found that CTSB, LAMP1 and STARD3 levels are increased in the liver of *Npc1^−/−^* mice (Figure 3C). Although we not used homogenates from primary cultured hepatocytes for the western blot analysis, it is likely that the changes observed in these experiments are from hepatocytes, since they account for approximately 78% of liver tissue volume.; while non-parenchymal cells constitute about 6.3%, of which about 2.8% are endothelial cells, 2.1% Kupffer cells, and 1.4% hepatic stellate cells [22,23].

**Figure 4:**
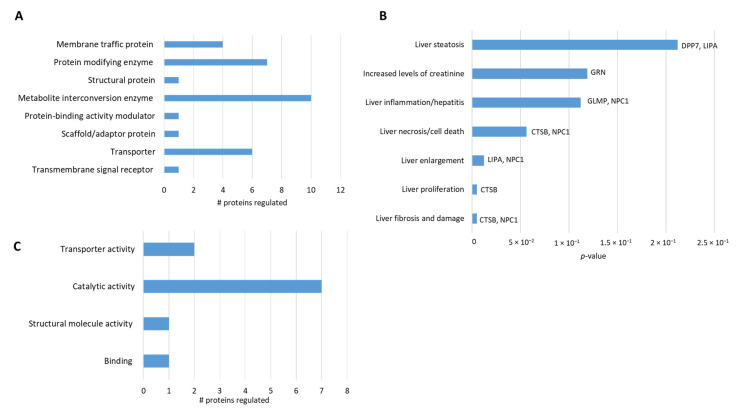
Analysis of differentially expressed lysosomal and mitochondrial proteins. (**A**) The differentially expressed 47 lysosomal proteins were mapped to GO categories (protein class). The bars represent the number of proteins associated with the respective terms. (**B**) The bar chart represents the lysosomal protein toxicity function filtered by hepatotoxicity. The bars represent the *p*-value and in each bar, the protein that participates in the process is labeled. (**C**) The 10 differentially expressed mitochondrial proteins were mapped to the GO categories (protein class). The bars represent the number of proteins associated with the terms.

Then, we classified lysosomal and mitochondrial proteins based on category and their role in hepatotoxicity. The lysosomal proteins are mostly enzymes, transporters, and membrane-associated proteins (Figure 4A). Among them, our analysis found proteins related to liver damage such as LIPA, DPP7, and GLMP (Figure 4B). Interestingly, we found that CTSB, a lysosomal protease, is upregulated and related to liver steatosis, fibrosis, and liver inflammation, suggesting that it might have a central role in liver damage in NPCD (Figure 4B). Among the mitochondrial proteins, most are enzymes (Figure 4C) and some of them, such as AKR1B10 [41] and VAT1 [42], have been previously connected with liver damage. Taken together, our results suggest that there is not only lysosomal but also mitochondrial dysfunction in NPC liver cells. These proteins might be new potential targets for the treatment of NPC hepatic disease.

## 4. Discussion

Our knowledge about LSDs and their pathophysiology is rapidly increasing but much remains to be learned. The understanding of pathological mechanisms is key for the identification of new therapeutic targets. To improve our knowledge on these diseases, new technologies, such as high-throughput approaches [43], are necessary.

NPC disease is an LSD that causes neurodegeneration and still poorly understood liver damage. To investigate the pathophysiology underlying liver damage in NPCD at the parenchyma cell level, we performed proteomic analysis of hepatocytes from *Npc1^−/−^* mice.

Our proteomic analysis revealed that the proteins with the most significantly altered expression patterns are related to liver damage, lipid metabolism, and inflammatory response. In addition, in the group of up/downregulated proteins, 47 proteins were identified as lysosomal and 10 as mitochondrial proteins. Importantly, we found that a subset of those proteins, CTSB, LIPA, DPP7, and GLMP had been previously connected with liver fibrosis, liver damage, and steatosis. Our report agrees with other studies that show that hydrolases expression is upregulated in liver tissue at the protein [20,44] and mRNA levels [45,46,47] in NPCD. Among these enzymes, cathepsins are the most abundant proteases in vertebrates. They perform essential functions in all living organisms controlling homeostatic and pathological processes [48]. Interestingly, our results show that some cathepsins are upregulated in NPC hepatocytes (CTSD (fold change = 3.58), CTSB (fold change = 2.63), CTSH (fold change = 3.70), CTSZ (fold change = 3.91) and CTSS (fold change = 5.16)). CTSZ has been shown to be markedly increased in hepatocytes from patients with later stages of cholestatic liver disease, particularly primary biliary cholangitis. Increased expression and altered localization of CTSZ were also observed in hepatocytes at end stages of other cholestatic liver diseases, including Alagille syndrome, obstructive jaundice, and sepsis [49]. In addition, CTSB [50] and CTSD [51] are altered in liver damage. In NPC mice, increased hepatic CTSD mRNA levels have been observed from the first week of life. Progressive increase in liver CTSD protein levels correlates with the age of the animals [47]. Interestingly, elevated serum levels of CTSD were found in NPC patients [47]. CTSD plasma levels show a positive association with the degree of NPCD severity in patients [47]. These results agree with our proteomic analysis in NPC hepatocytes, suggesting that CTSD could be a pathological marker of liver damage in NPCD.

CTSB has been described as a potential diagnostic biomarker for chronic liver disease [52]. Moreover, its inactivation decreases liver damage [53]. Interestingly, CTSB is increased and promotes liver fibrosis in a Niemann Pick type A (NPA) disease animal model [54]. This is a lysosomal disease similar to NPCD, suggesting CTSB as a therapeutic target that may be relevant in the treatment of liver fibrosis in related LSDs. Furthermore, CTSB has been described as a key molecule that mediates neurodegeneration in the NPA animal model [55]. Therefore, CTSB could be added to the list of common pathogenic characteristics of LSDs [56].

Alterations in the expression of GLMP [57,58] are related to chronic liver injury and DPP7 has been involved in apoptosis in hepatic cells [59]. Also, alterations in the expression of LIPA are involved in liver damage [60]. Among mitochondrial DEPs, AKR1B10 [41] and VAT1 [42] have also been implicated in liver damage. Nevertheless, there is no evidence linking them to liver damage in NPCD. Our results provide valuable information that complement other studies on lysosomal proteins such as GLMP (fold change = 2.62), LIPA (fold change = 2.94) and DPP7 (fold change = 3.41), as well as mitochondrial proteins (AKR1B10 (fold change = 2.54) and VAT1 (fold change = 2.98)) in liver damage and NPCD. However, we cannot rule out that the overexpression of these proteins in *Npc1^−/−^* hepatocytes may be the result of compensatory upregulation instead of a cause of injury during the development of liver damage in NPCD.

The differentially expressed proteins found in this study show that there are many proteins involved in liver damage that could explain some hallmarks of NPC liver disease. We detected some similarities with previous proteomic analyses performed in NPC mice.

Surprinsingly, we found a clear induction of CD68 expression which is an endo-lysosomal protein present in monocyte-derived cells. We can speculate that CD68 levels increase in NPC samples as a consequence of TFEB activation, analogous to what is observed in HeLa cells overexpressing TFEB. This same study showed that ablation of TFEB impedes CD68 mRNA expression [61]. However, we cannot rule out that the cultures could have some contamination.The method that we used to generate primary hepatocytes includes a centrifugation at low centrifugation speed. This step allows the isolation of hepatocytes from other non-parenchymal cells since they are heavier. In addition, we only found cells with hepatocyte morphology by optical microscopy.

Previous studies on liver tissue showed altered expression of other proteins linked to lysosomes, such as LAMP1 and v-ATPase complex subunits [20], among others, which agree with our results. Also, increased levels of lysosomal proteins, such as LAMP1, were reported in the cerebellum and cortex from 11 weeks-old *Npc1^−/−^* mice. In this case, the analysis was performed using AJS-ESI technology and label-free quantitative proteomics [44]. Moreover, recent proteomic profiling of NPC lysosomes shows proteolytic and structural defects [62].

Interestingly, similar results on transcriptome expression have been described in the liver of NPC mice [45,46,47]. Moreover, our analysis of canonical signaling pathways agrees with previous reports that show changes in pathways involved on iron metabolism, autophagosome formation/maturation, and autophagy in the liver [20], cerebellum, and cerebral cortex [44]. Nevertheless, we have not found signficant changes in LIMP2 expression in our proteomic analysis. LIMP2 was detected in our dataset and there was also some degree of upregulation in the NPC hepatocytes. Unfortunately, this upregulation was not statistically significant, unlike what other studies have shown [20,63]. However, these previous reports evaluated LIMP2 expression in liver homogenates. Moreover, van der Lienden et al. analyzed HEPG2 cells exposed to U18666A and they did not find changes in LIMP2 expression by western blotting. Therefore, LIMP2 overexpression could occur in liver cells other than hepatocytes in NPCD.

In addition, Pergande et al. 2019 showed increased expression of members of the fatty acid-binding protein family FABP5, in the cerebellum from *Npc1^−/−^* mice. Other studies also showed alterations in the expression of these proteins in the *Npc1^−/−^* cerebellum [64] and liver [20].

Interestingly, our proteomic analysis shows that one of the proteins with the most altered expression is a member of the fatty acid-binding protein family, FABP4. The role of FABP4 in hepatocytes and liver is relatively unknown. Its basal expression in the liver is low but highly inducible in response to different noxae, including ischemia/reperfusion injury, sepsis and hepatocellular carcinoma [65,66,67], suggesting that its expression could be increased in *Npc1^−/−^* hepatocytes in response to damage. Concordantly, previous studies from our group in NPC liver showed increased mRNA expression of FABP4 [46], confirming an alteration of cholesterol metabolism in the NPC liver.

We also found changes in the levels of specific lysosomal and mitochondrial proteins. Similar patterns of lysosomal and mitochondrial protein expression were found in a proteomic analysis from NPC1 human fibroblasts carrying the most common mutation in humans (I1061T) [68]. These results suggest that both organelles respond to lipid accumulation in NPCD. Several studies show mitochondrial alterations in NPC cells. These results imply that although accumulation of cholesterol in lysosomes and lysosomal dysfunction is the primary pathogenic event, changes in lysosomal-mitochondrial communication could induce secondary accumulation of cholesterol in mitochondria, triggering functional problems that contribute to disease progression [36]. Indeed, cells depleted of NPC1 show an increase in the number of membrane contact sites (MCS) between lysosomes and mitochondria. The lysosome-mitochondria MCS requires the lysosomal STARD3 protein. This protein binds cholesterol [69] and is elevated in NPC cells, as we show in this work and in previous studies from our group [40].

Interestingly, mitochondrial dysfunction has also been described in other LSDs such as NPA and Gaucher disease [56]. Therefore, mitochondrial and lysosomal alterations may contribute to the pathogenesis of these diseases. Understanding these mechanisms may open opportunities to search for new treatments.

We also know that ITGB2, which is overexpressed in *Npc1^−/−^* hepatocytes (Supplementary Table S2), participates in necrosis of liver cells [70], and its expression is necessary for liver injury mediated by neutrophils [71]. A higher expression of this protein in the NPC liver could explain the liver damage observed.

Nudt7 resides in the peroxisomes and is the major CoA-degrading enzyme in the liver [72]. Analysis of lipid metabolism in mouse liver indicates that this enzyme contributes to the regulation of fatty acids and bile acid metabolism in this organelle [73]. Its downregulation in NPC hepatocytes could be due to decreased peroxisomal fatty acid beta-oxidation, as has been observed in the liver from NPC mice [74]. On the other hand, it has been shown that MUP1 is secreted by the liver and regulates glucose and lipid metabolism [75]. Interestingly, there is evidence suggesting that MUP1 plays a relevant role in mitochondrial dysfunction, liver steatosis, and insulin resistance [75,76].

Catechol-o-methyltransferase (COMT) is decreased in NPC hepatocytes (Supplementary Table S2) and is an enzyme responsible for the metabolism of catechols, such as catecholamines and catechol estrogens. Liver COMT deficiency is linked to a disruption of glucose homeostasis in mice [77].

On the other hand, ANXA1 plays a functional role in modulating hepatic inflammation and fibrogenesis in a model of liver damage [78], with its overexpression being considered a protective factor. In NPCD, ANXA1 overexpression could be explained as a way to counteract liver damage produced by the accumulation of cholesterol.

In summary, using LC-MS/MS analysis we were able to find proteins whose expression is significantly changed in hepatocytes from *Npc1^−/−^* mice. Interestingly, these proteins are related to liver damage, lipid metabolism and inflammatory response. In addition, in the group of up/downregulated proteins, 47 proteins (30%) were identified as lysosomal proteins and 10 (7%) as mitochondrial proteins, suggesting that alterations in both organelles underlie relevant pathogenic mechanisms. Our findings may contribute to the search for new molecular targets for the treatment of liver damage in NPC disease.

## 5. Conclusions

In conclusion, to investigate the pathophysiology underlying liver damage in NPCD, we performed proteomic analysis of hepatocytes from *Npc1^−/−^* mice. We identified and reliably quantified a total of 3832 proteins: 416 proteins presented a *p*-value smaller than 0.05, of which 37% were considered DEPs. Our results suggest that there are not only lysosomal but also mitochondrial protein alterations in NPC liver. These proteins are associated with liver fibrosis, liver damage, and steatosis and might be new potential targets for the treatment of NPC hepatic disease.

## Data Availability

The mass spectrometry proteomics data have been deposited in the ProteomeXchange Consortium via PRIDE [30] partner repository, with the dataset identifier PXD026623 and 10.6019/PXD026623.

## Supplementary Materials

The following information is available online at https://www.mdpi.com/article/10.3390/cells10082159/s1. Table S1: List of the number of proteins, peptides, and spectra identified and quantified with a *p*-value ≤ 0.05; Table S2: List of the most differentially regulated proteins with log_2_ fold change > 1 and log_2_ fold change < −1; Table S3: List of the most differentially regulated proteins with log_2_ fold change > 2 and log_2_ fold change < −1 classified by locations and types; Table S4: List of the most differentially regulated lysosomal and mitochondrial proteins with log_2_ fold change > 1 and log_2_ fold change < −1.
