# Supplementary material for: Proteomic Analysis of Niemann-Pick Type C Hepatocytes Reveals Potential Therapeutic Targets for Liver Damage
Source: Cells. 2021 Aug 21;10(8):2159. doi: 10.3390/cells10082159 (PMC8392304; doi:10.3390/cells10082159)
Supplement: Supplementary file 1 [file cells-10-02159-s001.zip › Supplementary Table_3.pdf]

Table 3. The most differentially regulated proteins with log_2 fold change >2 and log_2 fold change <-1 classified by locations and types.

Symbol	Entrez Gene Name	Log2 Fold Change npc/wt	Location	Type(s)
Mup22	Major urinary protein 22	-2,829	Extracellular Space	transporter
NPC1	NPC intracellular cholesterol transporter 1	-1,934	Cytoplasm	transporter
NUDT7	Nudix hydrolase 7	-1,919	Cytoplasm	enzyme
Mup1	Major urinary protein 1	-1,360	Extracellular Space	transporter
COMT	Catechol-O-methyltransferase	-1,318	Cytoplasm	enzyme
HSD17B2	Hydroxysteroid 17-beta dehydrogenase 2	-1,240	Cytoplasm	enzyme
LAMP1	Lysosomal associated membrane protein 1	2,067	Lysosomal Membrane	Other
GNG2	G protein subunit gamma 2	2,083	Plasma Membrane	enzyme
ITGB2	Integrin subunit beta 2	2,089	Plasma Membrane	transmembrane receptor
ANXA1	Annexin A1	2,165	Plasma Membrane	enzyme
MMP12	Matrix metallopeptidase 12	2,172	Extracellular Space	peptidase
SLC43A2	Solute carrier family 43 member 2	2,234	Plasma Membrane	transporter
ATP6V0 D2	ATPase H+ transporting V0 subunit d2	2,280	Cytoplasm	transporter
LILRB4	Leukocyte immunoglobulin like receptor B4	2,300	Plasma Membrane	Other
FABP4	Fatty acid binding protein 4	2,323	Cytoplasm	transporter
SLC66A3	Solute carrier family 66 member 3	2,361	Other	Other
CTSS	Cathepsin S	2,371	Cytoplasm	peptidase
SLC37A2	Solute carrier family 37 member 2	2,375	Cytoplasm	transporter
S100A1	S100 calcium binding protein A1	2,659	Cytoplasm	Other
LEPROT	Leptin receptor overlapping transcript	2,744	Plasma Membrane	Other
CD200	CD200 molecule	2,890	Plasma Membrane	Other
